# The Influence of Vermicompost and Various Concentrations of Lead on the Enzymatic Activity of Sierozem Soils of Kazakhstan

**DOI:** 10.1155/2023/8490234

**Published:** 2023-02-01

**Authors:** G. A. Sainova, N. A. Kaliyeva, D. Kh Yuldashbek, A. D. Akbasova

**Affiliations:** Ecology Research Institute, Khoja Akhmet Yassawi International Kazakh-Turkish University, Turkestan, Kazakhstan

## Abstract

The influence of vermicompost and various concentrations of lead on the activity of hydrolytic (urease and protease) and oxidative-reducing (catalase and dehydrogenase) enzymes in the sierozem soil of Southern Kazakhstan (Turkestan region) was studied. Background unpolluted soils served as a control. The work shows a change in the enzymatic potential when vermicompost (8 t/ha) and lead are introduced into the soil in the concentration range of 16 to 160 mg/kg Pb. As a result of experimental studies, a decrease in the activities of catalase, protease, and dehydrogenase and, conversely, an increase in the activity of urease with an increase in the lead content in the soil system were revealed. Introduction of vermicompost (vermicompost) into the soil caused an increase in the activity of all studied enzymes and a decrease in the translocation ability of Pb. Inhibition of the process of translocation of lead into plants by vermicompost creates conditions for obtaining environmentally friendly agricultural products.

## 1. Introduction

As a result of numerous anthropogenic and natural factors, hundreds of thousands of chemicals enter the environment, a significant part of which accumulate in the soil system. Heavy metal compounds occupy a special place among them. They are not biodegradable and are toxic to all living organisms [1, 2].

Heavy metals have a negative impact on the composition and properties of the soil, which reduce its fertility. Under the influence of heavy metals, disturbances occur in the structure of populations of different types of microorganisms living in the soil environment. At the same time, there is a decrease in the number of individual, agronomically valuable groups of microorganisms synthesizing various enzymes [3–5]. Enzymes are natural catalysts of many soil processes and they play a key role in the mineralization of organic substances, and participate in the circulation of nutrients [6, 7]. Enzymatic activity indicates the degree of biochemical reactions and can serve as an important biological indicator for assessing the quality of soils contaminated with various toxicants [8–10]. Enzymatic activity can react quickly even to small changes in environmental conditions [11–13].

Over the past two decades, there has been a growing interest in using enzymes as monitoring tools to assess environmental pollution with toxic metals. The biological activity of the soil is estimated mainly based on the activity of four enzymes: dehydrogenase, catalase, urease, and protease. The study of the enzymatic activity of the soil is useful for assessing its chemical degradation. In this regard, the study of the effect of organic fertilizers on the enzymatic activity of soils contaminated with various concentrations of heavy metals is of certain scientific and practical interest [14–16].

The purpose of this work is to study the effects of lead and vermicompost on the activity of enzymes (catalase, urease, dehydrogenase, and protease) in sierozem soil.

## 2. Objects and Materials of Research

### 2.1. Objects of Research

Sierozem soils and meadow clover that were grown on them were the objects used for research.

### 2.2. Sampling

Soil and plant samples were taken from the agricultural fields of the Sauran and Otrar districts of the Turkestan region. In the control experiments, soils from the Karatau reserve were used, on which the negative influence of anthropogenic factors is limited. Soil sampling was carried out by the “envelope” method on a 10 × 10 m plot. The sample was averaged by “quartering.” After being brought to an air-dry state, plant roots were selected and sifted through a set of sieves for subsequent separation of a fraction with a certain particle size (*d* = 1 mm).

### 2.3. Variants of Experiments

To study the effects of vermicompost and lead on the activity of enzymes (urease, protease, catalase, and dehydrogenase), the experimental options are given in (Table 1).

The studied soils are characterized by the following indicators: physical clay (fraction <0.01 mm)–18.0–32.5%; mechanical composition–28.0–34.0%; humus content–0.6 to 2.0%; CO_2_ (in terms of carbonates) –3.8–5.6%; рН_КСІ_–6.10 ± 0.05; рН_Н2O_–7.45 ± 0.05; cation exchange capacity from 6.06–8.23 mg-eq/100 g; content of mobile forms of potassium (K_2_O) –6 mg/100 g, and phosphorus (P_2_O_5_) –20 mg/100 g.

Prior to the experiments, the granulometric composition of the soils was determined, which were selected from agricultural fields located near landfills (150 m from the sanitary protection zone) as well as from the territory of the Karatau Reserve, as shown in in Table 2.

The results of the study of the granulometric composition of soils indicate that they are depleted in finely dispersed fractions (physical clay) and enriched with fine sand particles. In soils with depth, the content of the silt fraction decreases (<0.001 mm). This fraction consists mainly of secondary clay minerals, humus, and organo-mineral substances. Fertility is associated with the content of the silt fraction in the granulometric composition of the soil, since secondary clay minerals determine the absorption capacity of soils and, along with humus, are the main sources of mineral elements in plants.

### 2.4. Simulation Experiment

Soil contamination with lead was carried out artificially by adding lead acetate (Pb(CH_3_COO)_2_ × 3H_2_O) at concentrations of 16, 80, and 160 mg/kg (by lead ions). Acetic acid salts were chosen for modeling soil contamination due to their good solubility and ability to quickly and completely interact with the soil mass. After the introduction of lead, the soil was incubated in plastic containers for 21 days at a temperature of 23 ± 2°C. Soil moisture was maintained by watering with distilled water. In the control experiments, the initial soils were used without application and with the addition of vermicompost but without containing lead. The vermicompost was obtained by an accelerated method of vermicomposting protected by a Russian patent by the authors of this article [17].

## 3. Methods of Research

### 3.1. Methods of Extraction and Analysis of Humus Acids

The method recommended by the International Humus Society was used to isolate humus acid preparations from soils (International Humus Substances Society, IHSS) [18]. In soil samples, the determination of the total organic carbon content was carried out according to the method of I. V. Tyurin, which is based on the decomposition of organic matter by potassium bichromate in an acidic medium [19]. The carbon content of the silty soil fraction was determined by the method of C. Gambardella and E. Elliott [20]. The group composition of humus was determined by the pyrophosphate expression method of Kononova and Belchikova [21].

### 3.2. Methods for Determining the Enzymatic Activity of Soils and Plants

The activities of hydrolytic (urease and protease) and oxidation-reduction (catalase and dehydrogenase) enzymes were studied in the soil. The enzymatic activity was determined in a threefold analytical repetition with an average sample. When calculating the final results, statistical data processing was carried out (arithmetic mean, arithmetic mean error, and coefficient of variation).

Urease activity was determined by the method of Romeyko and Malinskaya based on account of the amount of ammonia formed during the hydrolysis of urea. In this method, a Nessler reagent is used, which gives a colored complex with ammonia. Its concentration is determined by the photometric method using the device “Concentration photovoltaic photometer” (KPP-3-“ZOMZ”) of Russian production (JSC “ZOMZ,” registered at the address: Moscow region, Sergiev Posad, Red Army Avenue, 212 V) with a blue light filter at a wavelength of 400 nm and a working cuvette length equal to 10 mm, with the limits of the permissible basic absolute error of the wavelength set ±3 nm. Urease activity was expressed in mg NH_4_^+^/g^−1^ of soil 24 h^−1^ [22, 23].

Proteases (peptidhydrolases) catalyze the hydrolytic cleavage of peptide CO-NH bonds in proteins or peptides to form peptides of lower molecular weight or free amino acids. Protease activity was determined by A. Sh. Galstyan's method and expressed in milligrams of glycine per 1 g of soil for 24 hours [24].

Catalase activity in soils and plants was determined by the gasometric method of A. sh. Galstyan, based on the determination of the volume of O_2_ released during the decomposition of hydrogen peroxide. The activity of the catalase enzyme was expressed in ml of O_2_ released in 1 min from 1 g of soil/plant [24].

To determine the activity of dehydrogenase, colorless tetrazolium salts (2,3,5-triphenyltetrazolium chloride-TTC) were used as a hydrogen acceptor, which were reduced to red formazan compounds (triphenylformazan-TPF). Determination of dehydrogenase activity based on TPF was carried out by A. Sh. Galstyan's spectrophotometric method using a green light filter with a wavelength of 500–560 nm and a 10 mm cuvette. Dehydrogenase activity was expressed in ml of triphenylformazan (TPF)/10 g of soil every 24 hours [24].

### 3.3. Statistical Analysis

The tables presented in the paper show the arithmetic averages and their standard errors for two independent experiments. The data were processed by the method of variance analysis (ANOVA), and the Wilcoxon rank test for matching paired signs was used to assess differences in measurements. The *P* ≤ 0.05 was considered statistically significant for all analyses.

All data on the enzymatic activity of soils are given for air-dried samples and statistically processed in the Statistica 6.0 program. Numerical data are presented in the form of “average value ± standard deviation.”

## 4. Results and Discussion

### 4.1. Humus and Enzymatic Activity of the Soil

Many features and properties of the soil, including stocks of the most important nutrients and compounds, absorption capacity, and other properties depend on the qualitative and quantitative composition of humus substances. A distinctive feature of humus is its dynamism. Every year, fresh plant residues are involved in the cycle of biochemical transformation and there is a continuous process of formation of specific humus substances and decomposition processes. The totality of the processes of decomposition of plant residues from humification and the formation and decomposition of specific humus substances create a peculiar regime in the soil, conditioned by and associated with the acidity of the soil solution, redox potential, water, and thermal regimes.

Table 3 shows the results of studies characterizing the total carbon content and group composition of humus in the studied sierozem soils located from the landfills of solid household waste (SHW) of the villages of Temir and Maidantal at a distance of 100 to 500 m (sampling depth 0–25 cm). Humus reserves in soils of rural lands of Temir ranges from 13.75 to 27.0 t/ha and in Maidantal it ranges from 23.2 to 42.8 t/ha.

Table 4 shows the results of the group composition of humus in soils and their fractions (≤0.001 mm) taken as control (background) from the territory of the Karatau Reserve.

The analysis of the data obtained on the content and stock of humus in the soils of the control variant and adjacent to the landfills was carried out. At the same time, a decrease in the content and stock of humus in soils exposed to technogenic effects was revealed.

The ratio of C_HA_/C_FA_ usually characterizes the group composition of humus substances. It can be seen from the data in Tables 3 and 4 that a sharp change in the type of humus due to the impact of landfills was not observed. This indicates the dependence of the type of humus on the biological and climatic conditions of its formation. The group composition of humus at sierozem, located in the zone of subsidence of emissions from landfills, was typical for soils of this genesis.

According to the classification of Alexandrova [25], in uncontaminated sierozem soil, the type of humus corresponds to humate fulvate (C_HA_/C_FA_ = 0.6–0.8) and in contaminated-fulvate (C_HA_/C_FA_ < 0.6) (Tables 3 and 4). The change in C_HA_/C_FA_ under technogenic influence shows a reduction in the proportion of humic acids in the composition of humus, which should be considered a negative phenomenon.

**Figure 1:**
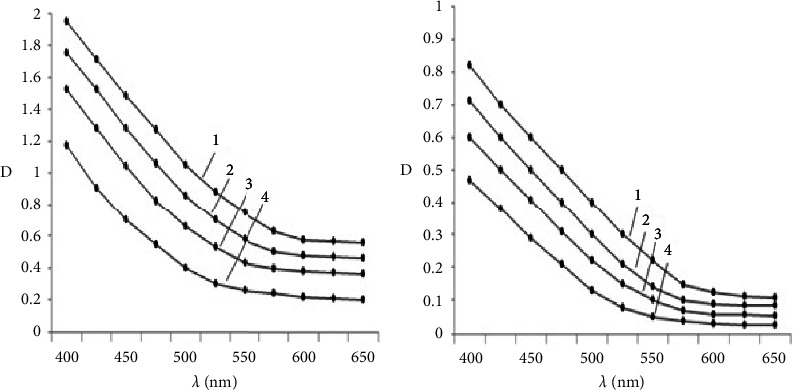
Optical density of humus acid. Humic acid (a) 1-control soil (without Pb); with the introduction of Pb into the soil, mg/kg: 2-16, 3-80, and 4-160. Fulvic acid (b) 1-control soil (without Pb); with the introduction of Pb into the soil, mg/kg: 2-16, 3-80, and 4-160.

To clarify the changes in the structural properties and nature of humus acids, electronic absorption spectra in the visible region were taken. Based on the dependence of the optical density (*D*) of humic acid and fulvic acid on the wavelength (*λ*), the values of the ratio of extinction coefficients *Е*_4_ : *Е*_6,_ characterizing the relative degree of condensation of humus acids were calculated (Figure 1).

The obtained spectrophotometric data also indicate a lower optical density of humus acid in soils subject to technogenic pollution. This to a certain extent indicates a decrease in the microbiological activity of the soil, the inhibition of the processes of “maturation” of humus substances, i.e., polycondensation processes. This, apparently, should also explain the decrease in the proportion of humus acid in the soil experiencing technogenic effects compared with the soil of the control variant. The main part of humus substances is represented by more active fulvic acids and relatively simple forms of humus acid.

The results obtained by us, i.e., the decrease in the total humus content caused by technogenic effects, as well as the revealed tendency to reduce the proportion of humic acids in the humus composition (Tables 3 and 4) are well explained from the standpoint of the general biothermodynamic theory of humification by Orlov [26].

### 4.2. Enzymatic Activity of Soils during Lead Contamination

**Figure 2:**
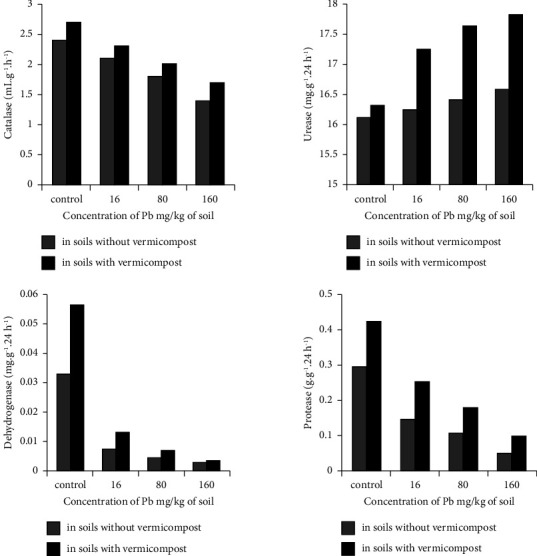
Dependence of enzyme activity on lead content in the soil system.

Figure 2 shows the results of studying the effect of various concentrations of lead on the activity of enzymes (catalase, urease, dehydrogenase, and protease) in a soil system that does not contain or contains vermicompost (8 t/ha).

The research results showed that lead-free sierozem soil, in terms of catalase enrichment, belongs to the poor (2.4 ml of oxygen per 1 min/g of air-dried soil). With an increase in the lead content in the soil, within the concentrations studied by us, a natural decrease in catalase activity is observed (Figure 2).

So, in comparison with the control, a decrease in catalase activity with lead content in the soil already occurs at a concentration of 16 mg/kg, which is 12.5%. A decrease in the value of catalase activity by 41.7%-at a concentration of the toxicant in the soil of 160 mg/kg.

Lead-free soil is very poor in terms of dehydrogenase enrichment (0.033 mg glucose/g day). With an increase in the lead content in the soil, there is a tendency to decrease dehydrogenase and protease activities under the influence of lead (Figure 2). When the concentration of lead in the soil is from 16 to 160 mg/kg, the activity for dehydrogenase decreases by 77.6–93.0% and for protease by 50.0–83.7%, respectively, compared with the control.

The soil uncontaminated with lead belongs to the average in terms of urease enrichment (16.1 mg of ammonia/10 g day). With an increase in the lead content in the soil, there is a tendency for a significant increase in urease activity (Figure 2). Thus, compared with the control, urease activity at a lead content in the soil of 160 mg/kg increases by 10.3%. Urease is an enzyme that catalyzes the hydrolysis of urea to ammonia and carbon dioxide. During the hydrolysis of urea by soil urease, local accumulation of ammonium ions occurs, which leads to an increase in the reaction of the medium to alkaline values. The alkaline environment promotes the formation of insoluble hydroxides, hydrocarbonates, carbonates, and other poorly soluble lead compounds. The course of these processes inactivates the action of lead; therefore, the catalytic activity of urease is maintained.

In all experiments, the introduction of vermicompost into the soil causes an increase in the activity of all enzymes, regardless of the concentration of lead. This can be explained by the fact that with vermicompost, microorganisms that contribute to the synthesis of enzymes are additionally introduced into the soil. The results obtained give an idea of how to increase the improved soil.

The results of the analysis of experimental data presented in Figure 2 indicate the identification of the clearest correlative relationship between the activity of catalase and the influencing factors, namely, the doses of lead and vermicompost in the soil. For other studied enzymes (urease, dehydrogenase, and protease), a similar dependence is not observed. The absolute value of the catalase enzyme activity indicators can be used for diagnostic purposes to characterize the ecological state of the soil system.

### 4.3. The Effect of Clover on the Activity of the Catalase Enzyme

According to the literature data, vegetation with a strong, deeply penetrating root system has a great influence on the enzymatic activity of soils [27, 28]. As an example, we have chosen meadow clover, which is a cosmopolitan plant species and is widely used as a fodder crop for animals.

Table 5 below shows the results of experimental studies to determine the activity of the catalase enzyme in soil treated with vermicompost (8 t/ha) and containing lead (from 0 to 160 mg/kg of soil), as well as clover grown in this soil. The soil taken from the arable layer of agricultural fields in the village of Shornak (Sauran district) was used for the experiment. The experiments were conducted in May–July 2020.

According to the results of the study, with an increase in lead concentration, a decrease in catalase activity in soil and clover during the first mowing is not observed. A sharp increase in catalase activity was revealed both in the soil and in clover in July (3rd mowing), when the formation of a developed root system occurs in the plant. A strong root system allows loosening of the soil, which in turn contributes to an increase in microbiological activity. The clover is rich in protein, enriching the soil with nitrogen thanks to symbiotic nitrogen-fixing bacteria appearing on its roots. As a result of an increase in the number of soil microorganisms, the enzymatic activity increases, and the rate of decomposition of hydrogen peroxide increases. Due to the improvement of the soil structure and the decomposition product of peroxide (Н_2_О_2_), oxygen enters metabolic processes and is released from the soil system into the atmosphere.

## 5. Conclusions

According to the research results, the dependence between the activity of soil enzymes (catalase, urease, dehydrogenase, and protease) in sierozem soil as a result of the impact of lead and vermicompost was revealed. Of all the studied enzymes, the dehydrogenase enzyme responsible for gas exchange processes turned out to be the most sensitive to Pb contamination. Its activity decreases by 77.6% and 93.0%, respectively, at concentrations of lead in the soil of 16 mg/kg and 160 mg/kg. A similar trend is observed with the protease enzyme. Protease activity decreases by 50.0% and 83.7%, respectively, at concentrations of lead in the soil of 16 mg/kg and 160 mg/kg. On the contrary, the activity of the urease enzyme, which is important for the nitrogen cycle, increases with an increase in the concentration of lead in the soil. Catalase activity can be recommended for use as indicators of lead contamination, since the degree of change in the activity of this enzyme directly depends on the dose of the contaminant and on the amount of humus substances.

## Figures and Tables

**Table 1:** Scheme of experiments by variants.

Soils for research	Lead content in soil samples (mg/kg)
Without adding vermicompost	0 (control)
	16
	80
	160

With the introduction of vermicompost (8 t/ha)	0 (control)
	16
	80
	160

**Table 2:** Granulometric (mechanical) composition of soils taken from the fields of rural districts of Sauran and Otrar districts of Turkestan region.

The place where samples were taken from fields near landfill	Sampling depth, in cm	Fraction sizes, in mm (their content, % of absolutely dry soil)						
		>3	3–1	1–0.25	0.25–0.05	0.05–0.01	0.01–0.001	<0.001
Maidantal (Sauran district)	0–5	1.5	2.1	0.9	20.1	32.0	6.4	13.6
	5–20	2.0	6.3	1.0	23.8	30.0	5.7	12.0
	20–30	2.3	4.8	2.1	29.6	35.2	6.1	12.4
	30–40	2.8	2.1	1.8	37.2	34.1	7.6	11.2
	40–50	3.3	3.7	1.3	28.9	29.8	6.6	10.8

Temir (Otrar district)	0–5	2.0	2.5	1.1	18.1	33.3	5.0	15.9
	5–20	2.4	4.3	2.0	22.6	31.2	5.8	13.1
	20–30	2.9	5.0	2.5	30.1	36.0	6.3	12.0
	30–40	3.1	3.0	2.3	32.2	33.0	7.3	12.2
	40–50	3.6	3.8	2.2	26.4	30.9	7.7	11.9

Karatau nature reserve (Sauran district)	0–5	5.0	3.2	5.0	33.4	28.1	10.6	14.3
	5–20	5.6	2.6	2.9	40.6	26.9	9.1	17.2
	20–30	1.8	1.7	3.3	30.0	21.4	7.8	17.4
	30–40	1.7	1.0	1.7	22.5	21.2	7.5	17.5
	40–50	2.0	2.0	1.2	18.7	20.5	4.4	20.2

**Table 3:** Group composition of sierozem humus (0–25 cm).

Sampling from fields at a distance (m)	Total carbon content (C_total_) (%)	The content of C (% of C_total_) in			С_HA_/С_FA_
		Humic acid (HA)	Fulvic acid (FA)	Nonhydrolyzable residue	
From the landfill “Temir” SHW (Otrar district)					
100	0.32	7.2	24.1	68.8	0.31
150	0.44	9.0	21.0	70.0	0.43
250	0.64	7.0	17.2	75.8	0.40
500	0.70	8.1	20.2	71.7	0.40

From the landfill “Maidantal” SHW (Sauran district)					
100	0.54	11.1	30.6	62.6	0.36
150	0.78	15.0	31.3	53.6	0.48
250	1.10	20.9	44.6	34.0	0.47
500	1.20	22.2	44.4	33.2	0.50

**Table 4:** Group composition of humus in sierozem soils of Karatau nature reserve and in its fractions (depth 0–25 cm).

Ordinary sierozem from the plots		Total carbon content (C_total_) (%)	The content of C (% on C_total_) in			С_HA_/С_FA_
			Humic acid (HA)	Fulvic acid (FA)	Nonhydrolyzable residue	
1	Soil	1.87	25.44	32.10	42.46	0.79
	Fraction ≤0.001 mm	1.55	29.87	51.00	19.13	0.59

2	Soil	2.14	30.06	37.20	32.74	0.81
	Fraction ≤0.001 mm	2.03	32.10	46.21	21.69	0.69

**Table 5:** Catalase activity in the soil-clover system.

The concentration of lead in the soil (mg/kg)	Catalase activity in soil (cm^3^) O_2_/g in 1 min		Catalase activity in clover (cm^3^) O_2_/g in 1 min		Total catalase activity (cm^3^) O_2_/g in 1 min (July)
	May	July	May (1st mowing)	July (3rd mowing)	
Plot no. 1					
Control (0)	2.8 ± 0.12	3.7 ± 0.15	10.4 ± 0.52	15.6 ± 0.75	19.3 ± 0.92
16	3.5 ± 0.13	5.0 ± 0.14	10.9 ± 0.50	11.6 ± 0.56	16.6 ± 0.74
80	4.0 ± 0.15	4.8 ± 0.17	13.2 ± 0.64	15.0 ± 0.73	19.8 ± 0.94
160	4.9 ± 0.11	6.4 ± 0.21	16.5 ± 1.0	22.8 ± 1.14	29.2 ± 1.33

Plot no. 2					
Control (0) + vermicompost	3.0 ± 0.16	3.2 ± 0.18	17.1 ± 1.10	20.9 ± 1.11	24.1 ± 1.22
16	3.6 ± 0.11	4.8 ± 0.13	18.9 ± 1.12	20.9 ± 1.16	26.7 ± 1.14
80	3.9 ± 0.15	7.3 ± 0.17	20.8 ± 0.87	19.9 ± 0.93	27.2 ± 1.26
160	4.7 ± 0.18	6.4 ± 0.21	21.8 ± 0.91	22.8 ± 1.14	29.2 ± 1.33

## Data Availability

The data used to support the findings of this study are included within the article.
